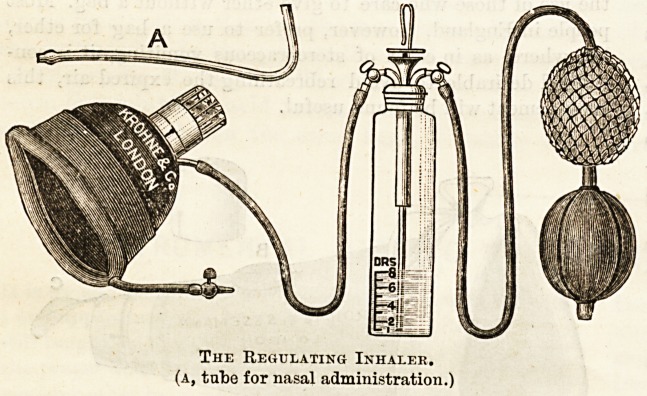# Instrumental Aids to the Administration of Anæsthetics

**Published:** 1896-10-24

**Authors:** 


### Instrumental Aids to the Administration of Anæsthetics.

It is by no means difficult to administer chloroform without any apparatus beyond a handkerchief or a towel, and it is still taught by some that this open method, as it is called, is the proper and best way to use it. It is possible also to administer ether by means of a towel rolled up in the shape of a cone; but it is difficult, and from the amount of ether used it is both expensive and disagreeable, while from the great coldness of the vapour the process is attended with risk to the patient. With nitrous oxide, on the other hand, it is impossible to produce proper anæsthesia without an apparatus.

We thus have three degrees of necessity, if one may so say, for the use of apparatus in the administration of anæsthetics, and it can hardly be doubted that a good deal of the popularity of chloroform, notwithstanding its admitted dangers as ordinarily given, has arisen from the fact that it has seemed easy to administer it without fuss or any parade of apparatus. At the same time, it is impossible to overlook the fact that this particular drug, which has been productive of so large a number of deaths, is the one which has been usually administered in such a manner as to preclude all possibility of knowing the quantity given or of estimating the strength of the vapour inhaled, and it seems not unreasonable to believe that the mortality from chloroform might have approximated much more nearly to that from ether if the dosage had been arranged with that accuracy which can alone be attained by the aid of instrumental methods of administration.

In regard to the three most commonly used anæsthetics, a moment's consideration will show that a different form of apparatus is required for each. It is clear that with chloroform it is the easiest thing in the world to get a sufficient quantity of the vapour, the danger being chiefly in the direction of giving too much; while in regard both to ether and nitrous oxide the special use of and necessity for an apparatus lies in the fact that without one it is next to impossible to get enough.

Of the various instruments which have been devised for the administration of chloroform some aim at providing means for regulating the dose of the vapour, others at leaving the access of air absolutely unimpeded, others at enabling the administrator to see the face of his patient, while others again aim at enabling him to observe the condition of the respiration and the pulse action of the heart while the process is going on. One of the great dangers of chloroform arises from its irregular administration and especially, according to some, from the occasional inspiration of an over strong vapour. When the towel is used this is likely to occur at the moment

The Precise Drop Bottle.

Skinner's Inhaler.

when, after a long pause, a deep respiration is taken; the vapour which has accumulated during the rest being drawn in at the first deep breath.

To obviate this Skinner's mask has done good service in the hands of many in all parts of the world. The principle on which it acts, when properly used, is that, the chloroform being dropped upon it at no greater rate than it is required, there is no reserve to go on evaporating, as there is in a folded towel which may hold perhaps a couple of drams of chloroform. Unfortunately too often Skinner's inhaler has been used merely as a neat and cleanly method, and the benefit of the mask has been considered to lie in its keeping the chloroform off the nose! Instead, then, of the drop by drop method, the flannel has been freely sprinkled with chloroform at variable intervals, and thus many of the evils of the towel, such as irregular administration, and the accumulation of a quantity of strong vapour over the mouth of the patient whenever respiration is suspended for a time, have been rather accentuated than removed. An efficient drop bottle then is an essential if chloroform is to be safely administered by a Skinner's mask, and the same applies to Esmarch's inhaler. General practitioners, who always have bottles and corks at hand, are apt to think that the common plan of cutting a nick down the side of the cork is good enough; and it

Vajna's Inhaler.
(Mayer and Meltzer, London.)

may be admitted that by making the nick to run only part way up the cork and then driving it in just to the point which will enable the chloroform to flow, a fairly efficient drop bottle can be easily made. But a very slight alteration in the position of the cork will upset the arrangement, and a properly constructed bottle, the rate of outflow from which can be graduated, is a great advantage.

Skinner's inhaler, however, has the great disadvantage of covering up the patient's face to a considerable degree. To obviate this a very useful inhaler has been devised by Dr. Vajna, the rim of the inhaler being made of glass and the flannel being stretched over the opening, which is sufficiently large to leave plenty of air way. It is to be noted, both in regard to this inhaler and the Skinner, that the flannel used should be very thin and open. The object of the flannel is not, in any way, to keep in the vapour, but merely to hold enough chloroform to be evaporated by one inspiration,

Silk's Transparent Celluloid Inhaler.

which is a very, very small quantity. The glass of the Vajna inhaler is easily cleaned, and the flannel is very readily changed. Another form of the same apparatus is made with a bunch of flannel projecting over the mouth for the use of those who care to give ether without a bag. Most people in England, however, prefer to use a bag for ether, but where, as in cases of stercoraceous vomiting, it is considered desirable to avoid rebreathing the expired air, this arrangement will be found useful.

The Hyderabad Cone.

With the same object of being able to see the face, Dr. Silk also has contrived an inhaler of transparent celluloid for use with the A. C. E. mixture, or with ether. Various forms of cones of leather or of celluloid, such as the so-called Hyderabad cone, have also been devised, the intention be that they should at first be held at some distance from the face, and then be gradually approximated. No doubt they are better than the towel, especially when they are provided with a free opening throughout, and with a feather indicator to show how the respiration is going on, but for prolonged anæsthesia they share with the towel the disadvantage of having to be re-charged, and thus causing irregular administration.

The Junker apparatus has a great advantage over all those in which the evaporation of the chloroform depends on the activity of the respiration. In it the amount of vapour produced is entirely under the control of the administrator. By

Carter Braine's Safety Junker.

means of a hand-ball air is forced through the liquid chloroform, and so soon as this is chilled by evaporation, the amount of vapour given off depends approximately upon the amount of air which is driven through it. Mr. Carter Braine has devised

The Regulating Inhaler.
(A, tube for nasal administration.)

an improvement of this instrument by altering the shape of the bottle in such a way as to prevent the possibility of the fluid chloroform being splashed up into the vapour tube, and to render it unnecessary to keep the bottle in the upright position during administration. These objects are also attained, although in a somewhat different way, by othe makers. Teske's modification of Junker's inhaler made by Maw, Son, and Thompson, contains some useful features.

Nicholson's Oxy-chloroform Inhaler.

Like those previously mentioned, it also is so arranged that there is no fear of liquid chloroform being projected upon the face in whatever position it is used. It is also provided with a tap so that the chloroform can neither be spilled nor wasted by evaporation when not in use, and when the tubes are removed it can be used as an ordinary drop bottle.

In Krohne's Regulating Inhaler the patient breathes into a face piece which has so large an opening into the outer air that no possible obstruction to respiration can be produced by it; this orifice is furnished with a feather indicator by which the slightest respiratory movement is made manifest, and thus the bellows may be compressed only during inspiration; and as the amount of chloroform can be regulated by the extent of the compression, it is possible to mix with the air drawn in at each inspiration just the amount of chloroform which it is desired to give. If desired a Skinner's mask can be fitted to this apparatus, and can thus be charged with vapour ready made instead of having chloroform dropped upon it. This arrangement does very well for self-administration and for midwifery, but for ordinary work the face piece with the feather indicator is better. Mr. Nicholson, of Liverpool, has sought to lessen the dangers of chloroform by giving oxygen along with it, but of the advantages of his arrangement we have no personal experience.

*To be continued.*

## Figures and Tables

**Figure f1:**
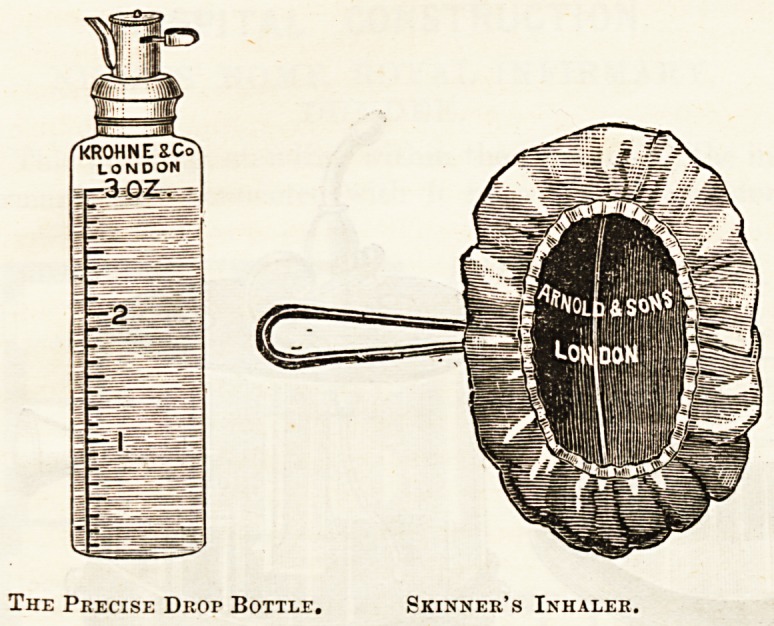


**Figure f2:**
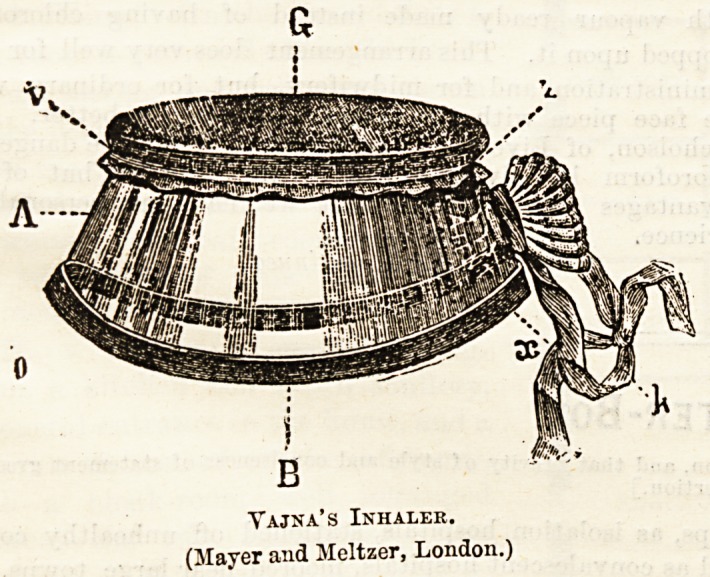


**Figure f3:**
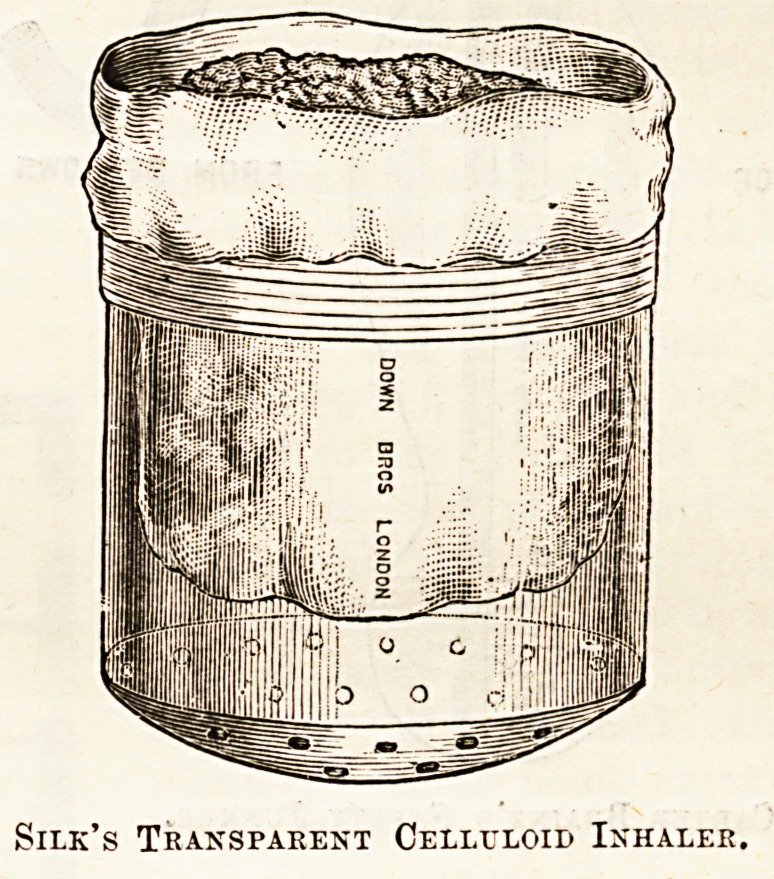


**Figure f4:**
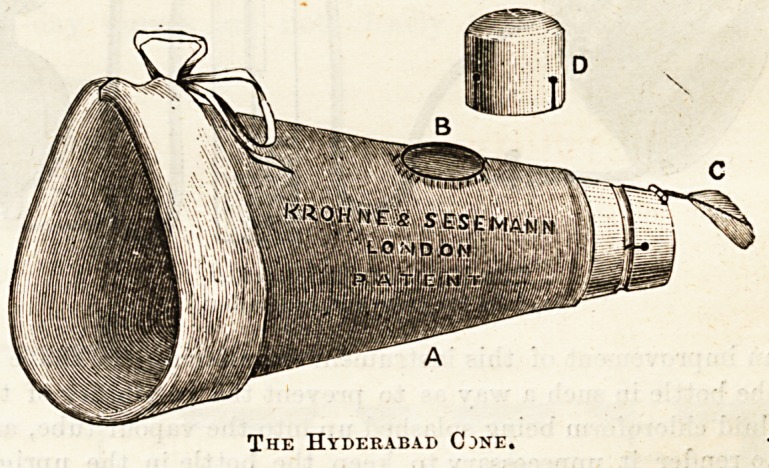


**Figure f5:**
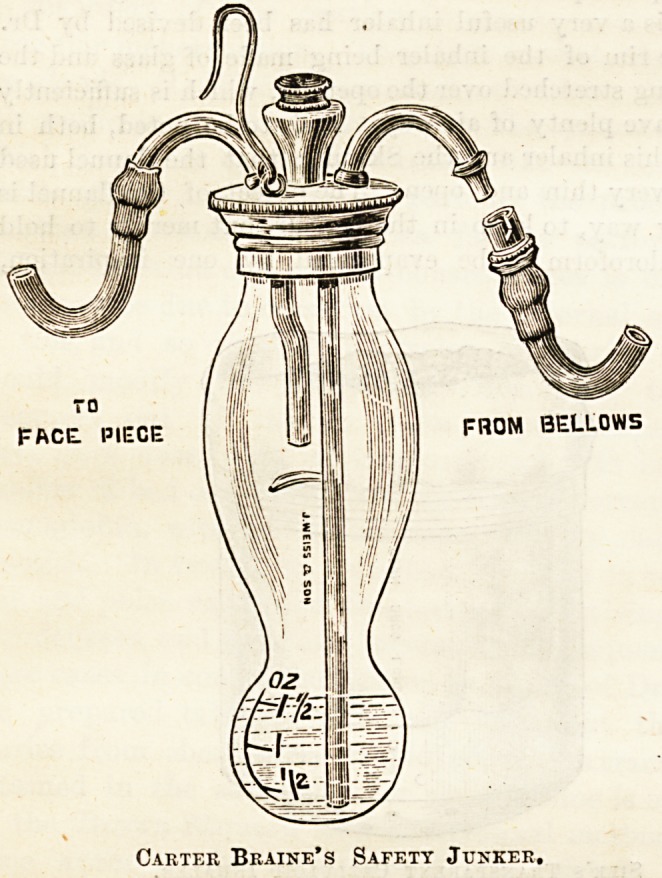


**Figure f6:**
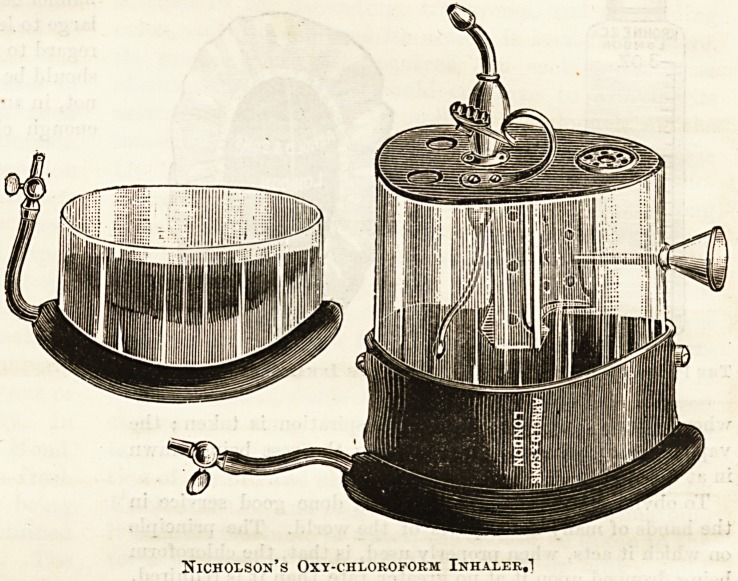


**Figure f7:**